# Dr. Lewis R. Palmer

**Published:** 1912-11

**Authors:** 


Dr. Lewis R. Palmer died in Rochester on October 2, 1912, aged 44 years. He was born and brought up in Clyde, N. Y., but lived and practiced in Baltimore and had returned to his home on a visit. Graduated Hahnemann Medical College, Philadelphia, 1892; formerly president of the Maryland State Homeopathic Society; president of the Homeopathic Hospital of Maryland, Baltimore; professor of diseases of heart and lungs in the Southern Homeopathic Medical College, Baltimore. He was buried in Clyde. He leaves his wife and one child. The cause of death was hemorrhage from gastric ulcer.

